# Clinical Presentation and Frequency of Metabolic Syndrome in Women With Polycystic Ovary Syndrome: An Experience From a Tertiary Care Hospital in Pakistan

**DOI:** 10.7759/cureus.11860

**Published:** 2020-12-02

**Authors:** Sarwat Anjum, Saima Askari, Musarrat Riaz, Abdul Basit

**Affiliations:** 1 Endocrinology, Baqai Institute of Diabetology and Endocrinology, Karachi, PAK; 2 Medicine, Baqai Institute of Diabetology and Endocrinology, Karachi, PAK

**Keywords:** pcos, metabolic syndrome, clinical presentation

## Abstract

Introduction: Polycystic ovary syndrome (PCOS) is a common endocrine disorder affecting women of reproductive age. The aim of this study was to determine the variations in the clinical presentation and frequency of metabolic syndrome (MetS) in women with PCOS.

Methods: This cross-sectional study was conducted at the Baqai Institute of Diabetology and Endocrinology, Baqai Medical University, Karachi, Pakistan, from April 2019 to March 2020. Women attending the endocrine clinic who satisfied the Rotterdam criteria of PCOS and agreed to participate in the study were included. Detailed personal and family history of menstrual cycle, hirsutism, diabetes, hypertension, dyslipidemia and obesity was noted along with measurement of vitals, anthropometric measures and calculation of the body mass index. Physical examination performed for signs of hyperandrogenism, insulin resistance and biochemical and hormonal evaluation was also carried out in recruited participants. Statistical analysis was done using the Statistical Package for the Social Sciences (SPSS) version 20 (IBM Corp., Armonk, NY).

Results: A total of 153 participants with mean age of 27.2±8.13 years were included in this study. Regarding clinical presentation, menstrual irregularity (oligomenorrhea 39.85%, amenorrhea 38.9%), followed by hirsutism 52.3%, was the most common presentation. Polycystic appearance of ovaries was noted in 33.3% of our study participants. MetS was identified in 46.4% participants (obesity was noted at the highest frequency at 82.4% followed by dyslipidemia at 56.2%).

Conclusion: We observed a high frequency of MetS in females presenting with PCOS. There is a need to evaluate women with PCOS for various components of MetS to prevent potential complications.

## Introduction

Polycystic ovary syndrome (PCOS) is one of the most common endocrine disorders that affects 5%-10% women of reproductive age [1,2]. It is clearly heterogeneous with speculative etiology, causing a wide range of reproductive, metabolic, endocrine and psychological effects. Among them, ovulatory dysfunction, menstrual irregularities, infertility, hyperandrogenism, increased insulin level, obesity, obstructive sleep apnea, nonalcoholic fatty liver disease, eating and mood disorders, cardiovascular disease (CVD) and an increased risk of type 2 diabetes mellitus (T2DM) are significant factors [3,4]. However, the most common presenting problem observed in PCOS is menstrual cycle disturbances (oligo/amenorrhea), hirsutism, infertility, dyslipidemia and metabolic disturbances due to insulin resistance (IR) [5]. Hyperinsulinemia, with consequent IR, has a significant role to play in its pathogenesis [6]. The diagnosis of PCOS is based on the presence of at least two of the three criteria, namely, chronic anovulation, hyperandrogenism (clinical or biological) and polycystic ovaries on ultrasound, according to Rotterdam criteria [2], which are widely used for the diagnosis of PCOS [6], in addition to excluding all other potential causes.

The prevalence of metabolic syndrome (MetS) is increasing throughout the world [7]. MetS and PCOS share a bidirectional relationship and common pathogenic factors that predispose women with PCOS to an increased risk of developing MetS [8]. Women with PCOS have a fivefold increase risk of developing MetS compared with women without PCOS, suggesting PCOS alone is an independent risk factor for MetS [9]. In one study, the reported prevalence of MetS was 44.6% in PCOS women [10]. MetS is a constellation of disorders that include abdominal obesity, impaired glucose tolerance/DM, hypertension and/or dyslipidemia [11]. MetS predispose patients with PCOS to high risk of CVD, T2DM and gynecological cancer [11]. PCOS and MetS share a bidirectional relationship and both conditions produce profound effect on fertility, reproductive biology and oxidative stress-induced vascular complications [8].

In a study conducted in Karachi, Pakistan, the reported prevalence of MetS in women with PCOS was 35.6%, which was significantly greater than the control group [12]. This substantial risk of increased morbidity and mortality has long-term implications for health of women with PCOS [13]. Data regarding the association of MetS and PCOS is scarce in our part of the world; therefore, our objective was to determine the clinical presentation and frequency of MetS in women presenting with PCOS at a tertiary care hospital [4].

## Materials and methods

This cross-sectional study was conducted at the Baqai Institute of Diabetology and Endocrinology (BIDE), Baqai Medical University, Karachi, Pakistan, over a period of one year from April 1, 2019, to March 31, 2020. All 13- to 45-year-old females attending the outpatient department satisfying the Rotterdam criteria of PCOS (defined as the presence of two of the following: oligo/anovulation, hyperandrogenism and/or polycystic ovaries on ultrasound) were recruited through non-probability consecutive sampling after taking informed verbal consent. Other causes of hyperandrogenism and menstrual irregularity were excluded by doing relevant lab investigations. Ethical approval was taken from the Institutional Review Board of BIDE.

Detailed personal, menstrual and family history of hypertension, diabetes, dyslipidemia and obesity (MetS) was taken on a predesigned proforma. MetS by definition comprises three or more of the following: (1) central obesity with waist circumference ≥80 cm (WHO cutoff for Asian women), (2) systolic blood pressure ≥130 mmHg or diastolic blood pressure ≥85 mmHg or taking antihypertensive medication, (3) fasting plasma glucose ≥100 mg/dl or previously diagnosed type 2 diabetes, (4) plasma high-density lipoprotein (HDL) cholesterol <50 mg/dl, (5) plasma triglycerides >150 mg/dl (as per American Heart Association/National Heart, Lung, and Blood Institute [AHA/NHLBI] criteria) [14]. Detailed physical examination was done including the measurement of blood pressure, pulse, waist circumference, and BMI. Signs of insulin resistance (acanthosis nigricans, skin tags) and signs of hyperandrogenism (hirsutism, acne, alopecia, clitoromegaly) were noted. The Ferriman-Gallwey score was used to assess the hirsutism (score >8 was considered positive). The biochemical and hormonal evaluation, including fasting plasma glucose, insulin, lipid profile, thyroid stimulating hormone, follicle stimulating hormone, luteinizing hormone, prolactin, estradiol, testosterone, 17-hydroxyprogesterone, dehydroepiandrosterone level, was carried out. The homeostatic model assessment of insulin resistance (HOMA-IR) was used to calculate insulin resistance: HOMA-IR = glucose fasting (mg/dl) x fasting insulin (µUI/ml) ÷ 405; values above 1.5 are indicative of insulin resistance. Pelvic ultrasound was performed for the evaluation of ovarian morphology.

Data analysis was done using the Statistical Package for the Social Sciences (SPSS) version 20 (IBM Corp., Armonk, NY) to compute mean, standard deviation, and percentages.

## Results

A total of 153 participants with a mean age of 27.2±8.13 years were included in this study. The mean age of menarche was 12.68±1.2 years while the mean BMI was found to be 31.68±7.37 (kg/m^2^). The baseline characteristics and biochemical parameters are mentioned in Table 1.

**Table 1:** Baseline characteristics and biochemical parameters of study participants HOMA-IR, homeostatic model assessment of insulin resistance; FSH, follicle stimulating hormone; LH, luteinizing hormone; TSH, thyroid stimulating hormone; IQR, interquartile range. Data is presented as mean±SD or n (%) or median (IQR).

Parameters	Mean±SD or N (%) or median (IQR)
N	153
Age (years)	27.2±8.13
Age at menarche (years)	12.68±1.2
Marital status	
Single	65 (43.9%)
Married	83 (55.1%)
BMI (kg/m^2^)	31.68±7.37
Waist circumference (cm)	86.71±16.72
Systolic blood pressure (mmHg)	116.37±15.14
Diastolic blood pressure (mmHg)	78.83±11.26
Fasting insulin	15 (10-24)
Fasting blood sugar (mg/dl)	96.35±29.4
HOMA-IR	4.71±4.11
FSH	5.3 (3.7-8.7)
LH	7.4 (4.9-14.4)
Estradiol	77 (33.4-155)
Testosterone	13.5 (1-57.6)
TSH	2.6±2.0
Prolactin	21.5±14.17

The most common clinical presentation of women with PCOS was menstrual irregularity; oligomenorrhea was reported in 61 (39.85%) and amenorrhea in 44 (38.9%), followed by hirsutism that was noted in 80 (52.3%) while acne and alopecia were found in 33 (21.5%) and 24 (15.6%), respectively. Moreover, infertility was present in 50 (32.6%) women, whereas 51 (33.3%) women had polycystic morphology of ovaries on ultrasound. The family history of diabetes, hypertension, dyslipidemia and obesity was present in 85 (55.6%), 65 (42.5%), 22 (14.4%), and 44 (28.8%) participants, respectively, as mentioned in Table 2.

**Table 2:** Clinical presentations of study participants PCOM, polycystic ovarian morphology. Data is presented as n (%).

Parameters	Frequency (n)	Percentage
N	153	-
Menstrual cycle irregularity	89	58.2
Dysmenorrhea	41	26.7
Oligomenorrhea	61	39.8
Amenorrhea	44	28.7
Dysfunctional uterine bleeding	17	11.2
Infertility	50	32.6
Hirsutism	80	52.3
Alopecia	24	15.6
Acne	33	21.5
Polycystic ovaries on ultrasound (PCOM)	51	33.3

**Figure 1:**
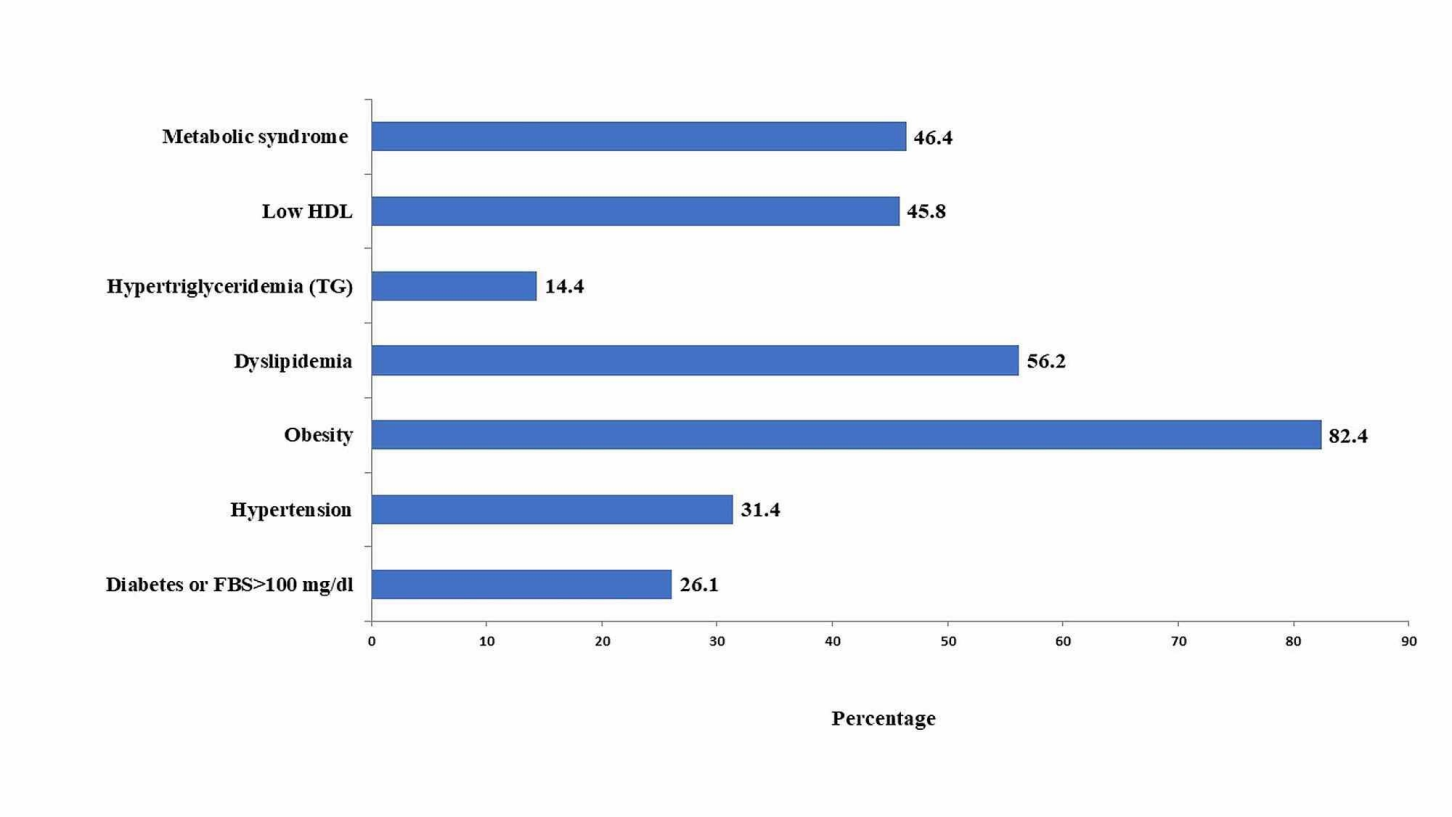
Frequency of metabolic syndrome in study participants HDL, high-density lipoprotein; FBS, fasting blood sugar.

The frequency and percentages of MetS and its individual parameters are shown in Figure 1. Out of 153 women with PCOS, 71 (46.4%) participants fulfilled the criteria of MetS. The obesity was noted in the highest frequency, in 126 (82.4%), followed by dyslipidemia, in 86 (56.2%), whereas 48 (31%) females had hypertension and 40 (26%) were either diagnosed with diabetes or impaired glucose tolerance.

Age, BMI, waist circumference and blood pressure had statistically significant association in PCOS women with MetS. The comparison among PCOS women with and without MetS in terms of baseline characteristics and lab parameters is shown in Table 3.

**Table 3:** Comparison of different variables in PCOS women with and without MetS PCOS, polycystic ovary syndrome; MetS, metabolic syndrome; HOMA, homeostatic model assessment; FSH, follicle stimulating hormone; LH, luteinizing hormone; TSH, thyroid stimulating hormone; IQR, interquartile range. Data is presented as mean±SD or n (%) or median (IQR). P-value <0.05 was considered to be statistically significant.

Variables	Without MetS	With MetS	P-value
N	82	71	-
Age (years)	25.63±7.5	29.01±8.5	0.01
Age at menarche (years)	12.82±1.21	12.51±1.17	0.146
Marital status			
Single	37 (44.21%)	28 (39.4%)	0.291
Married	40 (48.7%)	43 (60.6%)	
Not known	5 (6.1%)	0 (0%)	
BMI (kg/m^2^)	28.64±7.12	34.89±6.2	<0.0001
Waist circumference (cm)	75.97±17.99	93.29±11.9	<0.0001
Systolic blood pressure (mmHg)	110.3±12.55	122.43±15.16	<0.0001
Diastolic blood pressure (mmHg)	74.16±8.62	83.9±11.64	<0.0001
HOMA	4.47±3.58	4.88±4.48	0.693
Fasting blood glucose (mg/dl)	89.73±10.57	101.06±36.85	0.073
Fasting insulin	14 (9.2-19.8)	16.5 (10.6-25.25)	0.505
FSH	5.4 (4.2-8.7)	5.1 (3.5-8.5)	0.592
LH	7.2 (5.1-21.0)	7.5 (4.8-12.6)	0.367
Estradiol	74 (31.2-80)	165 (35-242)	0.222
Testosterone	20 (1-62.25)	7.5 (1.75-39.75)	0.782
TSH	2.4±1.76	2.7±2.28	0.616
Prolactin	24.43±17.15	19.65±11.86	0.254

**Figure 2:**
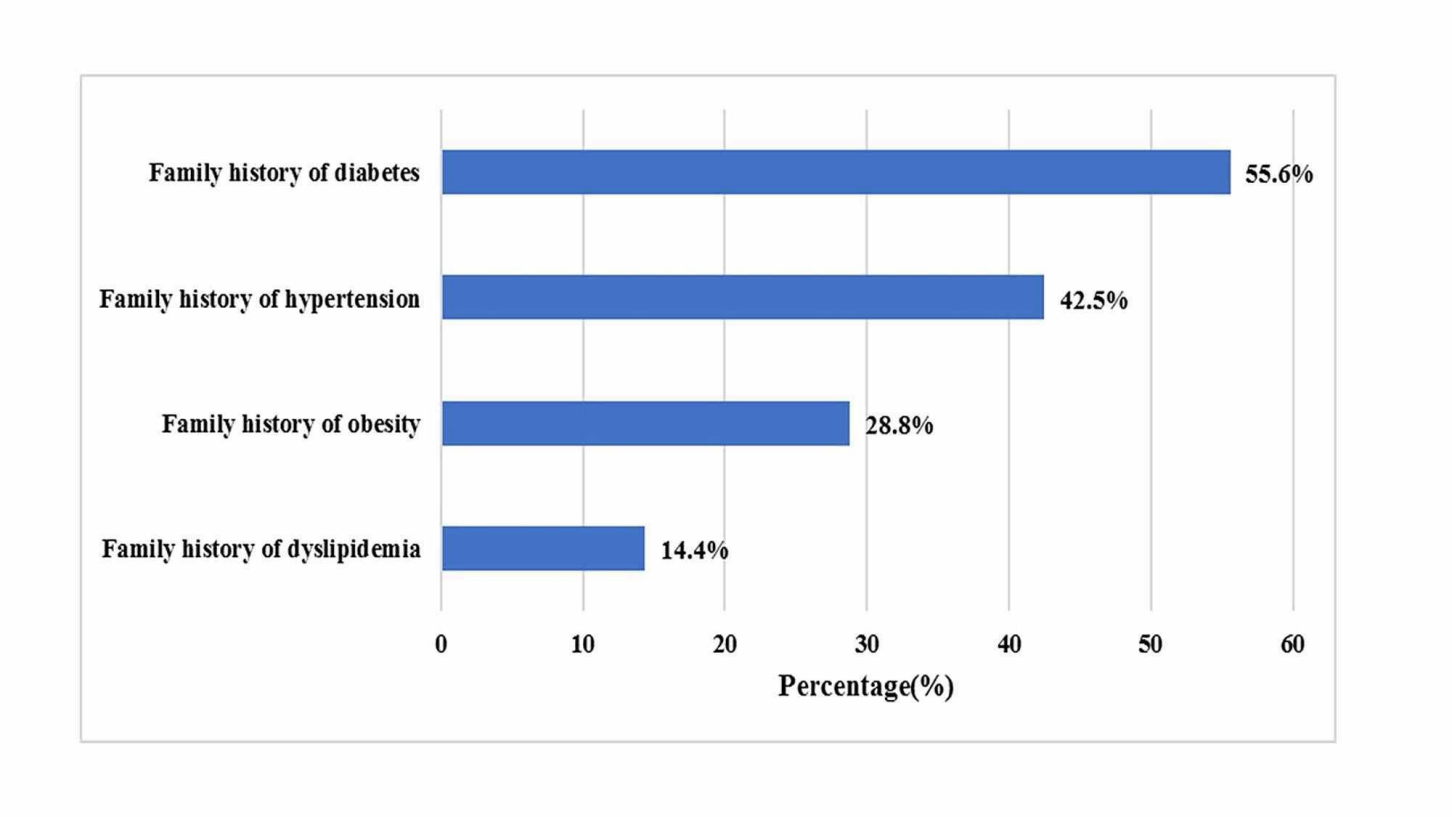
The components of metabolic syndrome in family members of women with PCOS PCOS, polycystic ovary syndrome.

As far as the individual component of MetS is concerned, diabetes was more prevalent in family members of women with PCOS followed by hypertension, obesity and dyslipidemia as shown in Figure 2.

## Discussion

The present study demonstrated a high frequency of MetS in women with PCOS. Among the different parameters of MetS, obesity and dyslipidemia were the two most frequent components in our study participants, while diabetes and hypertension were reported more in family members of the recruited women. The most common presenting complaint was menstrual irregularity (oligomenorrhea, amenorrhea) followed by hirsutism in our study participants.

The manifestation of MetS and clustering of its component varies in relation to studied population, ethnicity, and criteria applied to identify the MetS [12,15]. We observed MetS in almost half of our study participants; a similar finding was observed by Ehrmann et al., who reported a prevalence of 33.4% [15]. However, in contrast to our finding, MetS was noted only in 11.9% participants in another study [16].

Metabolic derangements are more obvious in obese as compared to non-obese PCOS counterparts [9]. Two-thirds of our study participants were obese; a similar prevalence was reflected in other studies [1,17]. In agreement with our observation, Anjum et al. [12] and Ehrmann et al. [15] observed that MetS and its distinct components are particularly common among females with increased BMI. However, Akram and Roohi [18] and Shanmugham et al. [19] reported obesity in only one-third of their study participants; this may be explained by the difference in the prevalence of obesity in different parts of the world [1]. The reported prevalence of obesity in Pakistan was 62.1% as per the second National Diabetes Survey of Pakistan (NDSP) during year 2016-2017 [20]. This high prevalence of obesity may be responsible for the increased frequency of MetS in our study population.

Women with PCOS, especially obese females, frequently displayed atherogenic dyslipidemia [10]. More than half of our study participants had dyslipidemia; the same was noted in another study [21]. In our study, low HDL contributed more as compared to high triglycerides to dyslipidemia, whereas Celik et al. [22] revealed significant high triglyceride levels in PCOS women as compared to controls. This disparity in data is enlightened by variation in the life style, dietary habits, prevalence of diabetes and obesity in different communities [10].

One third of our study participants were hypertensive, and similar finding was observed in another study [23]. In contrast to our finding, another study reported the frequency of hypertension in 10.7% of study participants [18]. Women with PCOS are at an increased risk of developing dysglycemia due to IR [5]. One fourth of our study participants had either impaired fasting plasma glucose or diabetes. Similar findings were revealed by other studies as well [24,25]. However, in contrast to our findings, Amato et al. noted impaired fasting glucose/T2DM in 12% of women with PCOS [26]; the observed diversity may be due to the variance in the prevalence of diabetes in different parts of the world.

The increased occurrence of different components of MetS among family members of women with PCOS pointed towards the genetic and environmental factors behind its pathogenesis. The significantly increased prevalence of components of MetS in the family history of our study participants is comparable with other studies also [18,27,28].

Menstrual cycle irregularities are noted early because of their monthly occurrence and may be the reason for being the common presenting complaint to health care professionals. In this study, menstrual irregularity was the main presenting complaint in the majority of study participants. This was also reflected in other studies [1,19,26]. In contrast to this finding, a meta-analysis revealed infertility and hirsutism as common presenting problem in females with PCOS [2].

Our study demonstrated hirsutism in almost half of PCOS women, and the same frequency was perceived by Amato et al. [26]. However, in contrast to our observation, Najem et al. [17] and Shanmugam et al. [19] found hirsutism in above 90% of study participants, whereas several other studies concluded that hirsutism affects 65%-75% of Southeast Asian women [1,21]. Statistics advocated that androgen level and hair follicle sensitivity to androgen are responsible for variations in the severity of the hirsutism seen in women with PCOS belonging to different ethnicities [5]. The number of women seeking medical advice regarding hirsutism varies widely depending upon the socio-cultural norms and acceptability in society.

Furthermore, a lot of women use herbal products and home remedies for hirsutism that may result in the variation of reported frequency of hirsutism.

Studies postulated that IR and compensatory hyperinsulinemia are common pathogenic factors for both PCOS and MetS [26]. In our study, IR was noted in two-thirds of women with PCOS and this finding is consistent with the research performed by Yu and Wangl [21]. Several other case control studies reported statistically significant high fasting insulin levels and HOMA-IR in women with PCOS [11,22,24]. However, this observation was contradictory to a study that showed IR in only one third participants [29]. The variation in genetic and environmental factors may be the reason behind the difference in severity of IR in different studied population [26]. Polycystic ovarian morphology (PCOM) on ultrasound was observed in one third of females with PCOS. In contrast to our findings, other studies demonstrated PCOM in more than 90 % of participants [19]. It is a well-known fact that ovarian androgenic dysfunction ranges from subclinical hyperandrogenemia in normal-variant PCOM to severe ovarian hyperandrogenism in most classic PCOS [3]. Moreover, polycystic ovaries are common in anovulatory women due to several causes and are not necessarily associated with PCOS [30].

Strength and limitation

This study provides local data on MetS in women with PCOS that would be helpful for further studies; however, this being a single-center study is the main limitation. Further studies are required to validate our findings.

## Conclusions

The frequency of MetS is high in females presenting with PCOS, and therefore, metabolic surveillance should be considered in such women to reduce the risk of potential complications.
